# Establishment of a mandible defect model in rabbits infected with multiple bacteria and bioinformatics analysis

**DOI:** 10.3389/fbioe.2024.1350024

**Published:** 2024-01-12

**Authors:** Yuan Zhao, Jun Su, Chong-yan Xu, Yan-bo Li, Tong Hu, Yi Li, Li Yang, Qiang Zhao, Wen-yun Zhang

**Affiliations:** ^1^ Department of Stomatology, 920th Hospital of Joint Logistics Support Force of People’s Liberation Army of China, Kunming, China; ^2^ Postgraduate Research Institute, Kunming Medical University, Kunming, China

**Keywords:** animal model, mixed bacterial solution, infectious mandible defects, bioinformatics analysis, the hedgehog pathway

## Abstract

**Objective:** A model of chronic infectious mandibular defect (IMD) caused by mixed infection with *Staphylococcus aureus* and *Pseudomonas aeruginosa* was established to explore the occurrence and development of IMD and identify key genes by transcriptome sequencing and bioinformatics analysis.

**Methods:** *S. aureus* and *P. aeruginosa* were diluted to 3 × 10^8^ CFU/mL, and 6 × 3 × 3 mm defects lateral to the Mandibular Symphysis were induced in 28 New Zealand rabbits. Sodium Morrhuate (0.5%) and 50 μL bacterial solution were injected in turn. The modeling was completed after the bone wax closed; the effects were evaluated through postoperative observations, imaging and histological analyses. Gene Ontology (GO), Kyoto Encyclopedia of Genes and Genomes (KEGG) pathway, and protein‒protein interaction (PPI) network analyses were performed to investigate the function of the differentially expressed genes (DEGs).

**Results:** All rabbits showed characteristics of infection. The bacterial cultures were positive, and polymerase chain reaction (PCR) was used to identify *S. aureus* and *P. aeruginosa*. Cone beam CT and histological analyses showed inflammatory cell infiltration, pus formation in the medullary cavity, increased osteoclast activity in the defect area, and blurring at the edge of the bone defect. Bioinformatics analysis showed 1,804 DEGs, 743 were upregulated and 1,061 were downregulated. GO and KEGG analyses showed that the DEGs were enriched in immunity and osteogenesis inhibition, and the core genes identified by the PPI network were enriched in the Hedgehog pathway, which plays a role in inflammation and tissue repair; the MEF2 transcription factor family was predicted by IRegulon.

**Conclusion:** By direct injection of bacterial solution into the rabbit mandible defect area, the rabbit chronic IMD model was successfully established. Based on the bioinformatics analysis, we speculate that the Hedgehog pathway and the MEF2 transcription factor family may be potential intervention targets for repairing IMD.

## 1 Introduction

The incidence of infectious mandibular defects (IMD) caused by war trauma and traffic injuries is increasing (Cicuendez et al., 2018), and these infections often result from a variety of bacteria; furthermore, traditional treatments are not effective, which seriously impacts the patient’s quality of life and the advances in implant restoration. The mechanism of mandible infection and the clinical problems caused by it have not yet been clarified, and a large part of this lack of knowledge is due to the lack of suitable animal models. For ethical reasons, it is difficult to carry out research on IMD in humans, so a suitable animal model of IMD is urgently needed to simulate clinical incidence and find key genes and pathways related to the development of IMD to provide a good research basis for addressing clinical problems.

There are many methods for preparing animal models of infectious bone defects. Lei MG et al. (Lei et al., 2017) implanted a stainless-steel tube soaked with bacterial fluid in the shin bone marrow cavity of rats and confirmed the signs of bone infection by testing. Tao J and Pearson JJ et al. (Pearson et al., 2020; Tao et al., 2020) first established defects in the shin and femur of rabbits, injected bacteria into the defect site, and successfully established a model of infectious bone defects. However, at present, the selected body parts for modeling are mainly conducted in limb bones, and the infection is usually caused by one bacterium. Furthermore, the osteogenic mechanism of the limb bones is different from that of the craniofacial bone, the traditional animal models of bone defect do not accurately simulate the clinically complex mandible infection, and there is still not an ideal animal model of compound bacterial infection in mandible defects.

In studying the occurrence and development of infectious bone defects, the effect of genetic polymorphisms on susceptibility to bone infection has been reported, which includes their effects on TNF-α, IL-1β, and IL-6; (Wang et al., 1985; Frangiamore et al., 2015; Hofmann et al., 2016a); these studies also showed the correlation between specific genes and bone infection. However, these studies only propose correlations at the genetic level and do not further investigate specific mechanisms. With the development of high-throughput technology, technologies such as DNA microarrays or transcriptome sequencing have become powerful tools for screening disease-causing genes by studying the differences in gene expression profiles between experimental and control groups, and the molecular function (MF), biological process (BP), cellular component (CC) and signaling pathways enriched in genes related to the occurrence and development of diseases have been identified.

**FIGURE 1:**
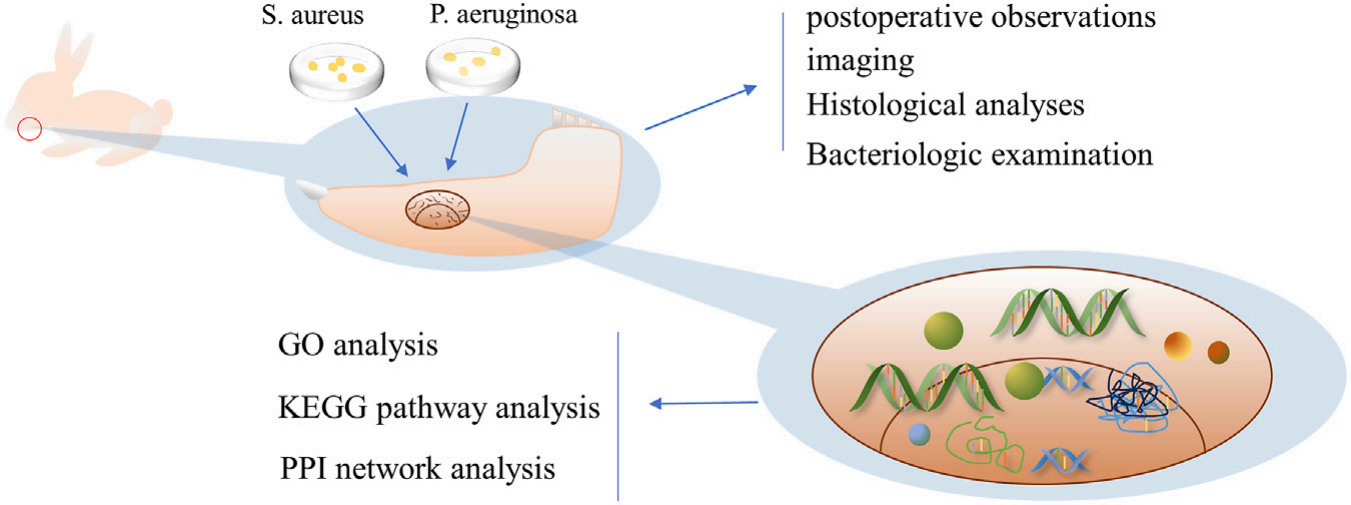
Schematic diagram of the research process Legend: In this study, the mandibular bone defect of rabbits was first established, and then the prepared bacterial solution was injected to successfully establish the IMD model, and bioinformatics analysis was conducted by transcriptomic sequencing.

In this study, to simulate IMD caused by war injuries, a mixture of *S. aureus* and *Pseudomonas aeruginosa* was used to establish IMD by animal experiments. And bioinformatics methods were used to analyze transcriptome data from animal models, identify DEGs, and analyze the functions, related pathways, core modules and core genes related to IMD (Figure 1); these results provide a direction for future research to repair IMD.

## 2 Materials and methods

### 2.1 Materials

A total of 28 New Zealand white rabbits (male and female, Conventional Animal) aged 3 months and weighing 2–2.5 kg were selected for the experiment (Kunming Chu Shang Technology Co., LTD. License Number [SCXK (Yunnan) K2019-0003]). The animals were raised in the Medical Animal Experimental Center of the 920th Hospital of the Joint Logistic Support Force [SYXK (Yunnan) K2020-0006], reared separately and given free access to feed and water for 1 week. All animals were handled in accordance with ethical regulations and were examined and allowed by the 920th Hospital Ethics Review Committee of the PLA Joint Logistic Support Force [2023–127 (Section) −01]. *S. aureus* (ATCC 29213), and *P. aeruginosa* (ATCC 27853, Luwei Technology Co., LTD. Shanghai, China) strains were obtained, as well as 5% sodium morrhuate (Xinyi Jinzhu Pharmaceutical Co., LTD. Shanghai, China, specification: 2 ML, 0.1 g/branch).

### 2.2 Methods

#### 2.2.1 Bacterial cultivation

*S. aureus* and *P. aeruginosa* were inoculated on Luria-Bertani (LB) culture plates (Solarbio, Beijing, China), incubated in a constant temperature incubator at 37°C for 36 h, and then removed. An appropriate amount of colonies were selected in 15 mL of LB culture medium (Solarbio, Beijing, China) for larger cultures. The mixture was incubated in a constant temperature incubator at 37°C for 36 h and diluted with sterile normal saline. The concentration of bacteria was adjusted to 3 × 10^8^ CFU/mL, and the two bacterial solutions were mixed and used immediately.

#### 2.2.2 Surgery

**FIGURE 2:**
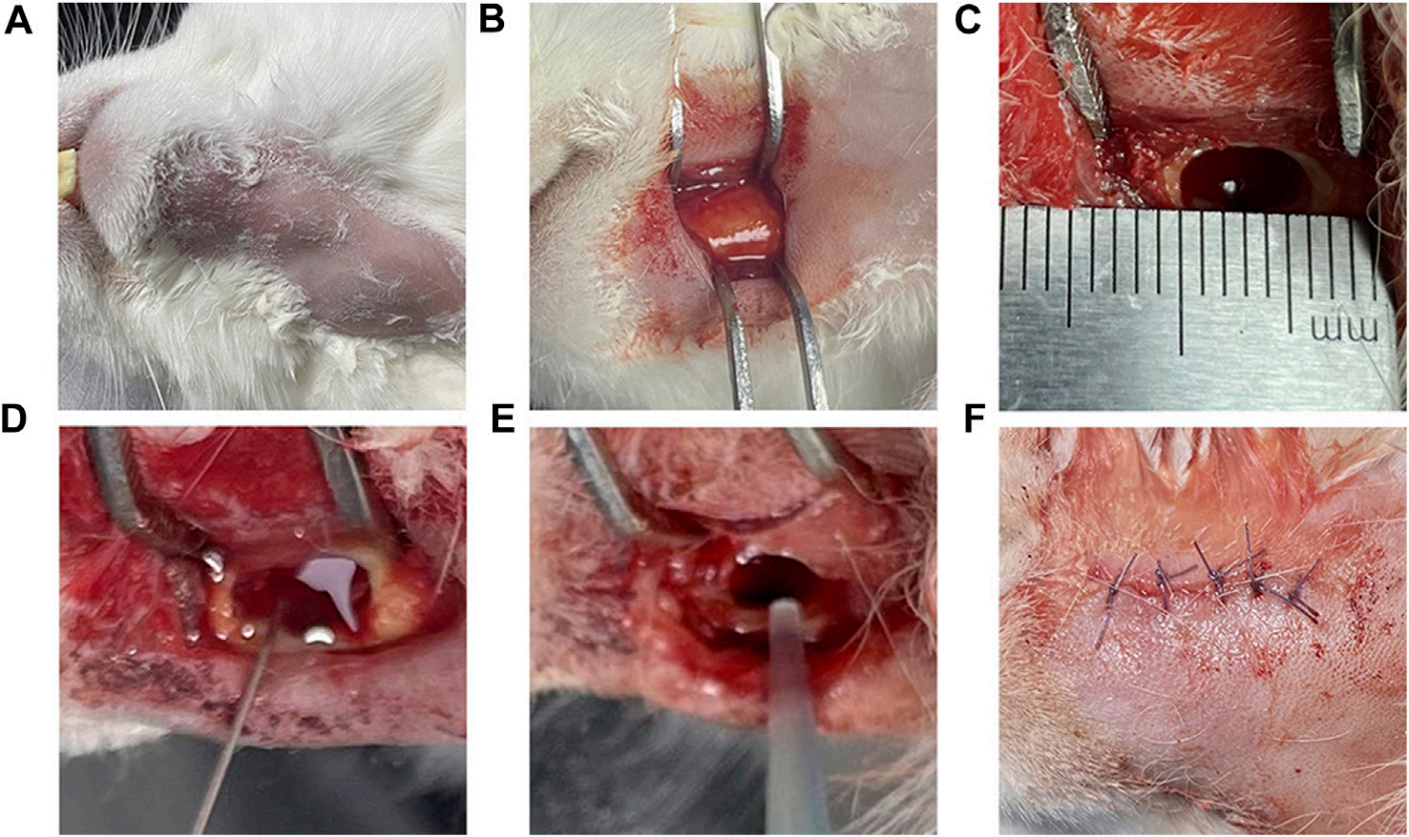
Model of the surgical procedure Legend: **(A)** Skin preparation and iodophor disinfection of the operative area. **(B)** The surgical area was dissected and separated the lateral surface of the mandible. **(C)** A 6 × 3 × 4 mm rectangular bone defect was prepared; and **(D)** 0.1 mL 5% sodium morate was injected and maintained for 5 min. **(E)** After rinsing with normal saline, 0.1 mL of 3 × 10^8^ CFU/mL mixed bacterial solution was injected; and **(F)** the bone wax was used for closure and the area was sutured layer by layer.

The experimental rabbits were weighed, and 3 wt% pentobarbital (Tengshan Biotechnology Co., Ltd. Kunming, China) was extracted and injected into the auricular vein for general anesthesia. After the anesthesia took effect, the head was fixed, and the skin of the mandible was prepared on both sides to facilitate follow-up observation of hair growth. In the iodoprene disinfection area, a 3–4 cm incision was made along the lower margin near the median union of the mandible, and the lateral bone surface of the mandible was exposed by blunt separation layer by layer. A circular bone drill with a diameter of 3 mm was used to drill holes, and a ball drill was used to trim the shape of a rectangular bone defect of 6 × 3 × 4 mm. A total of 0.1 mL of 5% sodium maphuate was injected into the bone defect, and after 5 min, normal saline was used for rinsing, 50 μL of 3 × 10^8^ CFU/mL mixed bacterial solution was injected, bone wax (Johnson & Johnson, Shanghai, China) was used to seal the defect, sterile normal saline was used for rinsing, and the wound was sutured layer by layer (Figure 2). After the operation, the rabbits were fed in cages without antibiotics. After the operation, rabbits were divided into two groups, and samples were collected at 14 and 28 days. Healthy rabbits fed conventionally for 1 month without any treatment were used as the blank group.

#### 2.2.3 Evaluation methods

Serious infection outside the experimental area, systemic blood-borne infection, extremely altered mental state, and difficulty eating were used as humane endpoints, and the experimental rabbits were selected for modeling. The experiment was conducted using a before-after study with the same samples.

##### 2.2.3.1 Temperature and food intake

The anal temperature of rabbits from 5 days before surgery to 5 days after surgery was measured at 9 a.m. every day, and the food intake during the same period was determined; the changes before and after surgery were analyzed.

##### 2.2.3.2 Wound healing

Skin healing at the incision was observed to determine whether there was sinus formation after modeling.

##### 2.2.3.3 Imaging examination

On the 14th and 28th day after modeling, a CBCT (Bondent, Shanghai, China) scan was performed on rabbits under anesthesia to observe the bone defect at the modeling site.

##### 2.2.3.4 Gross specimens

The rabbits were sacrificed by excessive anesthesia on the 14th and 28th days after modeling. The mandible on the modeling side was removed, and the soft tissues were removed to observe whether there was pus and new bone formation in the defect area. Animal carcasses were handled uniformly according to laboratory requirements.

##### 2.2.3.5 Histological analysis

The mandible was fixed with 4% paraformaldehyde (Solarbio, Beijing, China) and then decalcified in EDTA (Solarbio, Beijing, China) decalcification solutionuntil a fine needle was inserted into the bone cortex without resistance; then, paraffin-embedded sections were cut. Hematoxylin and eosin staining, Goldner’s tricolor staining (Source leaf, Shanghai, China) and tartrate acid fast phosphatase (TRAP) staining (Solarbio, Beijing, China) were performed and sections were viewed under a microscope. Goldner staining procedures were as follows: First, paraffin sections were dewaxed to water, and then Goldner staining solution A was mixed with Goldner staining solution B in equal proportion for nuclear staining. The sections were then stained with Goldner dye C, Goldner dye D, Goldner dye C, and Goldner dye E in turn. Finally, neutral gum sealed the section. TRAP staining procedures were as follows: First, paraffin sections were dewaxed to water. The slices were fixed using TRAP fixative. After rinsing sections with distilled water, add TRAP incubation solution and re-stain with hematoxylin. Finally, the sections were sealed with water-based sealer. Improved smelter scoring (Smeltzer et al., 1997) (Table 1) was used for the quantitative analysis of the HE section. The minimum score for each section was 0 points, the highest score was 4 points, and the total score was 16 points. Areas of the same size in TRAP-stained sections were randomly selected to count osteoclasts.

**TABLE 1:** Histological parameters and scoring system.

Score	Scoring rules
Intraosseous acute inflammation	
0	Not present
1	Minimal to mild inflammation with no intramedullary abscess
2	Moderate to severe inflammation with no intramedullary abscess
3	Minimal to mild inflammation with intramedullary abscess
4	Moderate to severe inflammation with intramedullary abscess
Intraosseous chronic inflammation	
0	Not present
1	Minimal to mild chronic inflammation with no significant intramedullary fibrosis
2	Moderate to severe chronic inflammation with no significant intramedullary fibrosis
3	Minimal to mild chronic inflammation with significant intramedullary fibrosis
4	Moderate to severe chronic inflammation with significant intramedullary fibrosis
Periosteal inflammation	
0	Not present
1	Minimal to mild inflammation with no subperiosteal abscess formation
2	Moderate to severe inflammation with no subperiosteal abscess formation
3	Minimal to mild inflammation with subperiosteal abscess formation
4	Moderate to severe inflammation with subperiosteal abscess formation
Bone necrosis	
0	No evidence of necrosis
1	Single focus of necrosis without sequestrum formation
2	Multiple foci of necrosis without sequestrum formation
3	Single focus of sequestrum
4	Multiple foci of sequestra

##### 2.2.3.6 Bacteriological analysis

Secretions from the defect were mixed in normal saline aseptically and coated on LB culture plates to observe whether there was colony growth. Afterward, specific primers (Table 2) were selected for different colonies for PCR (Solarbio, Beijing, China) detection to identify whether the infection was caused by the inoculated bacteria.

**TABLE 2:** List of specific primers.

GENE		Sequence (5,→3′)	Length	Tm	GC%
*S. aureus*	Forward Primer	GGGATGGCTATCAGTAA	17	43.88	47
*S. aureus*	Reverse Primer	TGAATCAGCGTTGTCTT	17	45.73	41
*P. aeruginosa*	Forward Primer	TACCTTCCTGTTTTGAG	17	43.95	41
*P. aeruginosa*	Reverse Primer	ATC​CAA​CTT​GCT​GAA​CCA​G	19	51.1	47

#### 2.2.4 Transcriptomic sequencing

Healthy rabbits were fed in the same environment for 1 month, and then a mandible defect model of rabbits infected with complex bacteria was established according to the described method. Fourteen days after the operation, 3 rabbits in the complex bacterial infection group and 4 rabbits in the healthy blank control group were sacrificed, and the mandibles were removed and stored in liquid nitrogen for quick freezing. Afterward, RNA libraries were constructed and sequenced to determine gene expression.

#### 2.2.5 Differential expression analysis

To compare the differences in gene expression between different samples, we used the R package edgeR (Robinson et al., 2010), which allowed for the identification of DEGs between the experimental group and the control group. The screening threshold was set as an adjusted *p*-value (adj.p) < 0.05, and genes whose fold change (FC) absolute value was greater than 2 were considered significant DEGs. The significant DEG expression levels of all samples were determined, and a heatmap of DEGs was drawn using pheatmap.

#### 2.2.6 GO term and KEGG pathway enrichment analysis of DEGs

For GO annotation for DEGs, the genes corresponding to a specific GO annotation were counted and then classified and plotted according to MF, CC, and BP (Ashburner et al., 2000). The genes corresponding to GO annotation were enriched, and the significant enrichment results were screened according to the threshold *p*-value < 0.05. All GO enrichment results were sorted according to *p*-value from smallest to largest, and the top 20 GO enrichment terms were depicted in bubble maps. The biological pathway information from the pathway analysis was derived from KEGG. First, KEGG annotation was performed for DEGs; then, the number of genes corresponding to KEGG annotation was statistically analyzed (Kanehisa and Goto, 2000), and functional classification was performed. The genes corresponding to the map number annotated by KEGG were enriched, and the significant enrichment results were screened according to the threshold *p*-value < 0.05. All KEGG enrichment results were sorted according to *p*-value from small to large, and the top 20 enrichment pathways were depicted in bubble maps.

#### 2.2.7 PPI network and module analysis

The DEGs were imported into the STRING database to convert protein names, and then the PPI network diagram and interaction information were obtained. Information on protein interactions were imported into Cytoscape software to create visual networks. Then, the nodes, degrees, betweenness centrality and edges in the visual network were analyzed. Using the MCODE plug-in to filter modules, the analysis parameters were set to node score cutoff = 0.2, degree cutoff = 2, K-core≥2, and max. depth = 100. The key nodes in the PPI network were identified using cytoHubba plug-ins. The cytoHubba plug-in uses the DMNC analysis strategy to predict and evaluate important nodes, and the nodes with the top 10 scores were considered core genes.

#### 2.2.8 Predicting transcription factors

Transcription factors were predicted using the iRegulon plug-in in Cytoscape software. The plug-in used the normalized enrichment score (NES) to assess the confidence of the predicted results. The greater the NES value is, the higher the confidence. In this study, transcription factors with NES>4.5 were used establish the network.

#### 2.2.9 Statistical analysis

The mapping software Image-Pro Plus, GraphPad Prism 9.0 (Graphpad software, Lajolla, CA, United States) and the statistical software SPSS 29.0 (IBM corporation, Armonk,NY, United States)were used for analysis. Data are expressed as x ± s. We used Paired T tests and one-way analysis of variance for preoperative and postoperative comparisons, and *p* < 0.05 was considered a significant difference.

## 3 Results

The mental state of animals was altered after modeling, and no death or loss occurred within 4 weeks. All 28 rabbits were observed during the experiment.

### 3.1 Changes in body temperature and food intake

**FIGURE 3:**
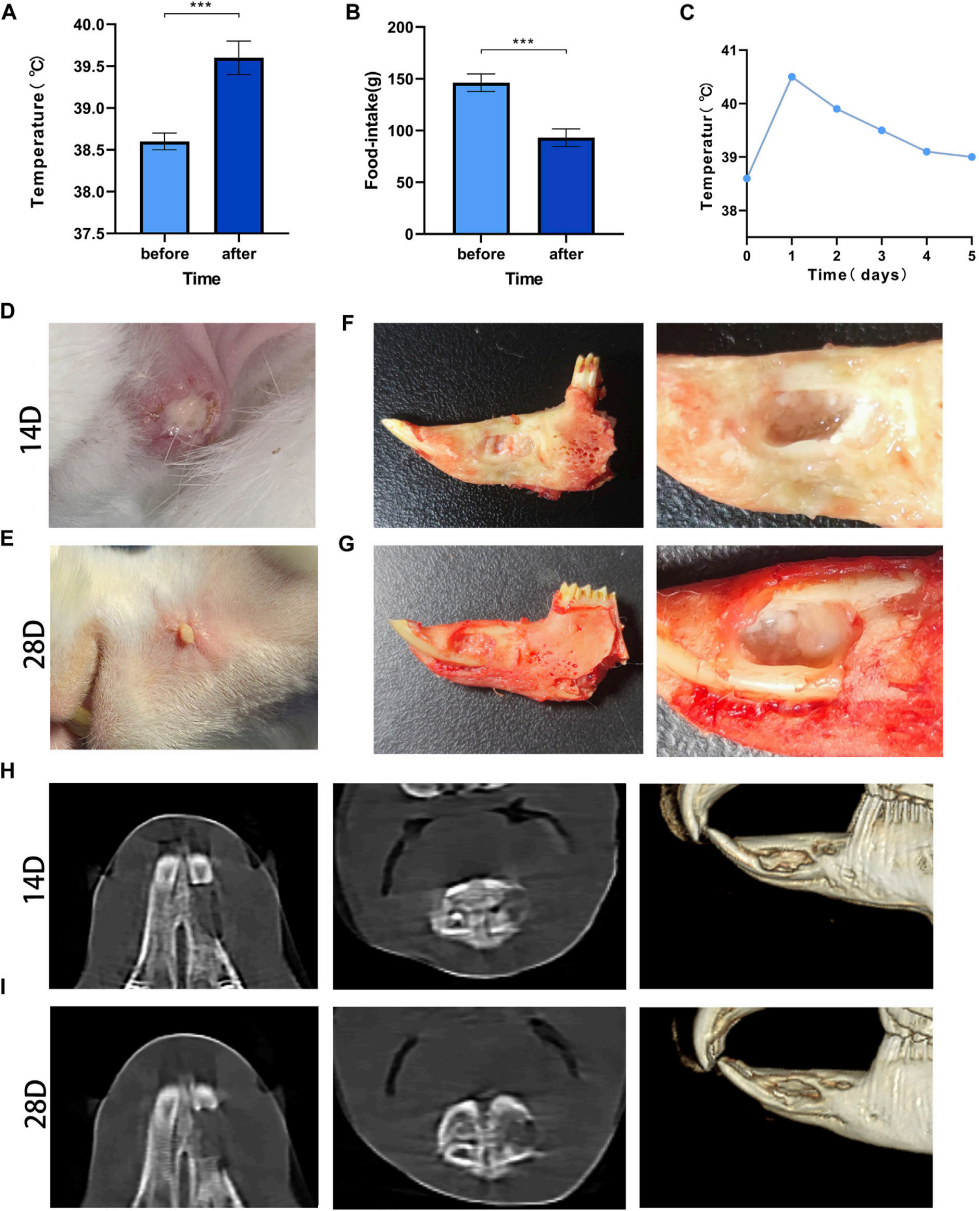
Post-modeling monitoring results and imaging results Legend: **(A)**. The difference in body temperature before and after modeling. **(B)**. The difference in food intake before and after modeling. **(C)**. The temperature changes before and after modeling. *** means *p* < 0.001 (n = 28). **(D)**. The result of wound healing at 14 days after surgery; **(E)**. The result of wound healing at 28 days after surgery. **(F)**. Gross specimens at 14 days after modeling. **(G)**. Gross specimens at 28 days after modeling. **(H)**. The results of imaging changes at 14 days. **(I)**. The results of imaging changes at 28 days.

The mean value of the measured data was determined, and statistical analysis showed that the body temperature was 38.5°C ± 0.1°C before modeling and 39.6°C ± 0.2°C after modeling, and the food intake decreased from 146.3 ± 8.5 g before to 93.1 ± 8.4 after modeling; the differences were significant (*p* < 0.001) (Figures 3A,B). The changes in body temperature reverted over time (Figure 3C).

### 3.2 Wound healing

All rabbits had local skin redness 3 days after modeling, subcutaneous swelling of different degrees and purulent secretions at the incision on day 14 (Figure 3D). The local condition of the rabbits improved somewhat before day 28, but there were still abscesses and sinus, and there was no significant increase in body hair when compared with the contralateral hair (Figure 3E).

### 3.3 Imaging changes

On day 14, the edges of the bone defect were blurred, and there were signs of bone destruction. Osteolysis, mainly manifested by decreased bone density, appeared in the bone marrow cavity, and the bone trabeculae’s number were decreased (Figure 3H). At 28 days, erosion at the edge of the defect was observed, the bone density at the distal end of the defect was significantly reduced, and the normal structure of the bone trabecula was disrupted (Figure 3I).

### 3.4 Gross specimens

The rabbits were sacrificed by excessive anesthesia at 14 and 28 days. After incision of the skin, fistulas were found, and small pus cavities were found in the soft tissue of the wound. After the soft tissue was removed, yellow‒white pus filled the bone defect area, the defect was not healed, and the pus was aspirated sterilely for bacterial culture (Figures 3F,G).

### 3.5 Bacteriological results

**FIGURE 4:**
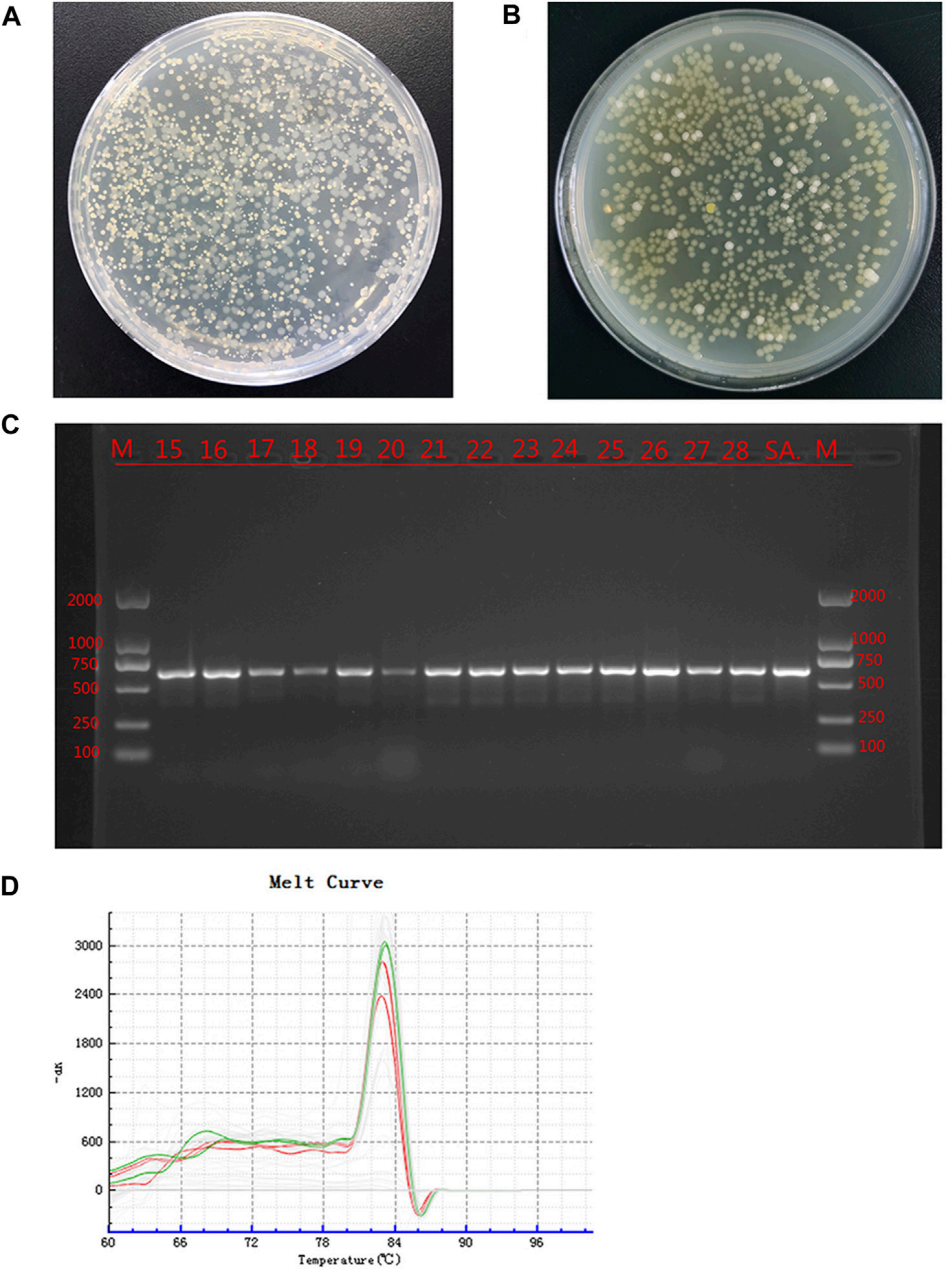
Bacterial culture and identification Legend: **(A)**. The bacterial culture of the secretions of defects at 14 days. **(B)**. The bacterial culture of the secretions of defects at 28 days. **(C)**. PCR results of the suspected SA sample and the SA standard strain, where M is the standard band, SA is the standard *Staphylococcus aureus* band, and the rest are the suspected *Staphylococcus aureus* samples. **(D)**. qPCR results of suspected PA samples and PA standard strains. Green is the dissolution curve of PA standard bacteria, and red is the dissolution curve of suspected PA. The curve trend and peak value were consistent.

The bacterial culture of secretions from the defects of all experimental animals were positive on day 14, and two types of colonies with different forms were visible; the cultures were still positive on day 28 (Figures 4A, B). The two colonies were selected aseptically for qualitative detection, and the suspected *S. aureus* samples and *S. aureus* standards were amplified by PCR using specific primers. The results showed that all samples and standard strains were amplified with clear bands of 618 bp (Figure 4C). At the same time, the suspected *P. aeruginosa* samples and *P. aeruginosa* standards were tested by qPCR, and the results showed that all the samples were positive (Figure 4D); the CT value was 24.34 ± 2.28. The above results showed that SA and PA were the bacteria found in the culture of secretions from bone defects.

### 3.6 Histopathological results

#### 3.6.1 HE staining results

**FIGURE 5:**
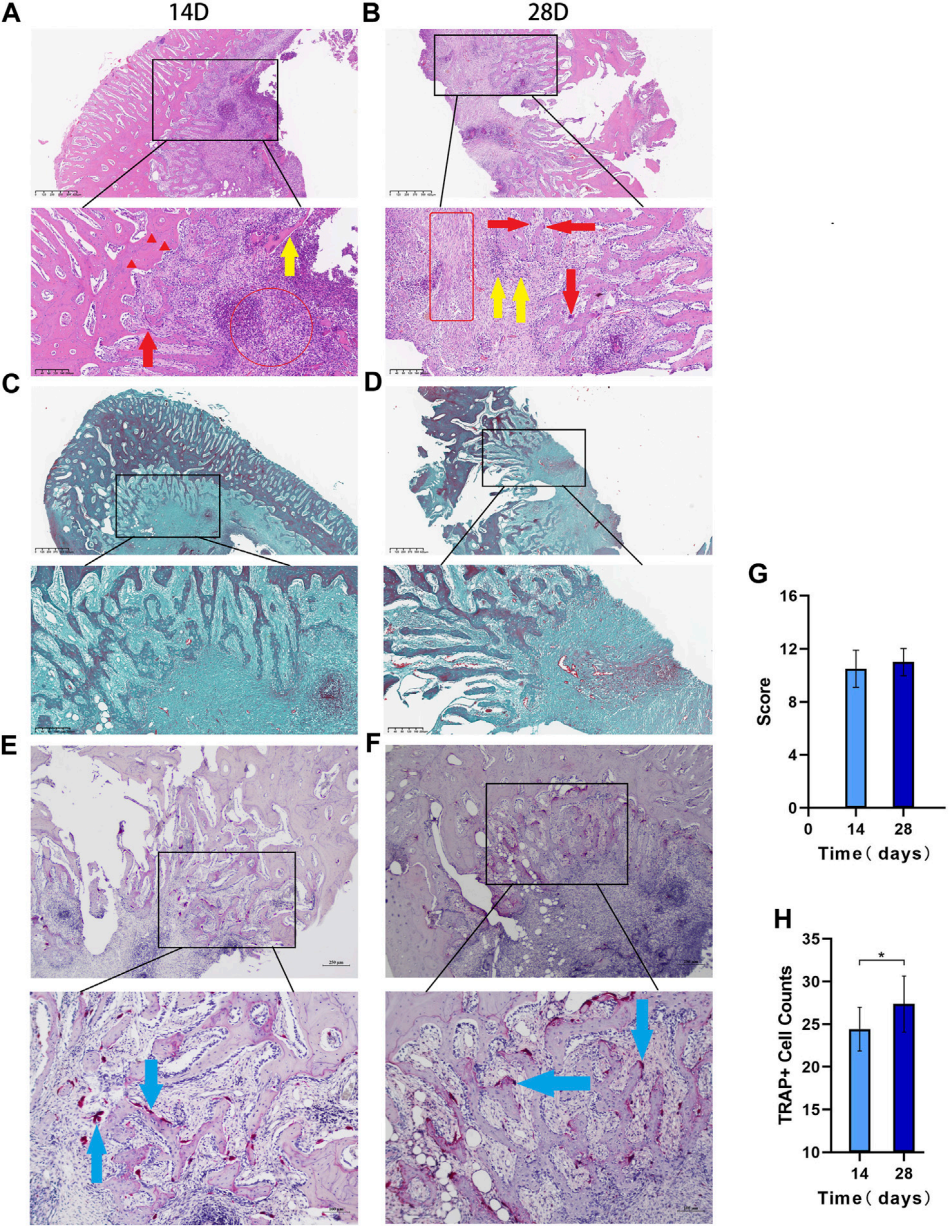
The histopathological results Legend: **(A)**. HE 14 days after modeling and **(B)**. 28 days after modeling, and the red circles in a and bindicate acute inflammation. The red box indicates chronic inflammation, the red arrows indicate macrophages, the red triangle indicates the loss of bone nucleus, and the yellow arrow shows free dead bone. **(C)**. Goldner staining at 14 days after modeling; **(D)**. Goldner staining at 28 days after modeling. **(E)**. TRAP staining results at 14 days and **(F)**. TRAP staining results at 28 days. **(G)**. Differences in the Smeltzer score were determined according to the Smeltzer scoring rules; the pathological changes between the two groups have no significant difference (*p* > 0.05, n = 14). **(H)**. Analysis of osteoclast count per unit area in the two groups. The blue arrows are osteoclast-positive areas (* indicates *p* < 0.05 and n = 14).

HE staining showed that on day 14, there was inflammatory cell infiltration, the bone trabecular structure was destroyed, abscesses were formed around the lesion, and osteolysis was manifested by disappearance of the bone nucleus (Figure 5A). On day 28, bone destruction became more obvious, and many inflammatory cells infiltrated the bone marrow cavity; furthermore, the number of macrophages increased and necrosis and abscesses formed, indicating aggravation of the bone infection. According to Smeltzer scoring rules (Table 1), the pathological changes between the two groups have no significant difference (*p* > 0.05, Figures 5B,G).

#### 3.6.2 Goldner staining results

Goldner staining showed that the bone cancellous around the defect was reduced on day 14 (Figure 5C), and bone cancellous damage in the medullary cavity was serious on day 28, which was consistent with the HE results. The trabecular structure of the bone at the edge of the defect was seriously damaged, the infection showed signs of spreading, and some new bone had formed (Figure 5D).

#### 3.6.3 TRAP staining results

The TRAP staining results showed that the osteoclast-positive areas were mainly clustered near the defect on day 14 (Figure 5E), and osteoclast activity and function were significantly increased on day 28 (Figure 5F). The number of osteoclasts per unit area significantly differed between the two groups (*p* < 0.05, Figure 5H).

### 3.7 Results of differential expression analysis

**FIGURE 6:**
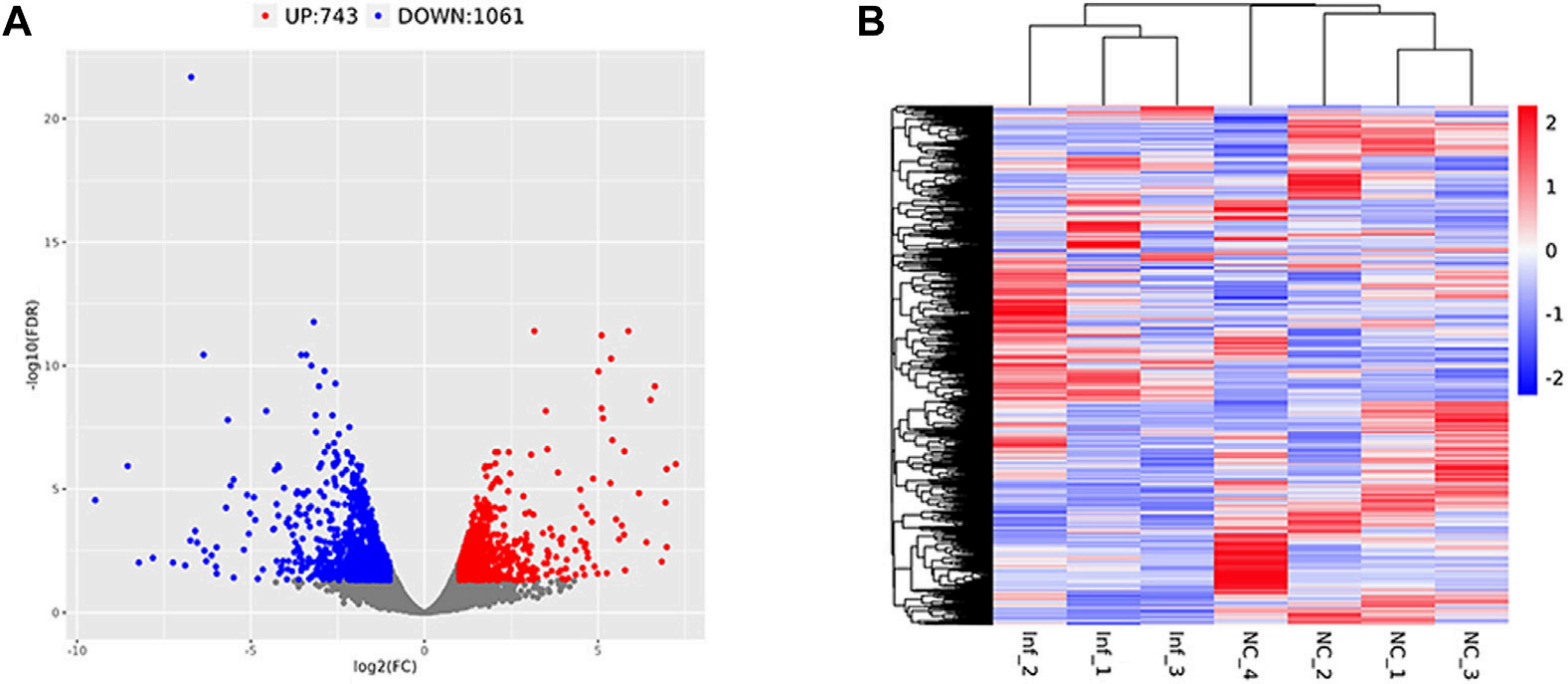
Differentially expressed gene volcano plot and heatmap Legend: Red represents upregulated genes, blue represents downregulated genes, and gray represents non-differentially expressed genes. **(A)** The differentially expressed gene volcano plot. **(B)** The differentially expressed gene heatmap.

Based on the criteria of adj. *p* < 0.05 and FC absolute value greater than 2, 1804 DEGs were identified after comparing the infected group and the control group,; among these, 743 were upregulated and 1,061 were downregulated (Table 3; Figure 6A). A heatmap was used to cluster DEGs and show the dynamic change in DEGs expression; red indicates upregulated expression and blue indicates downregulated expression. The expression changes and clustering results of DEGs in the test and control group are shown (Figure 6B).

**TABLE 3:** Number of DEGs.

SampleID	Total	UP_num	DOWN_num
INF_vs._NC	1804	743	1,061

### 3.8 The results of GO analysis of DEGs

**FIGURE 7:**
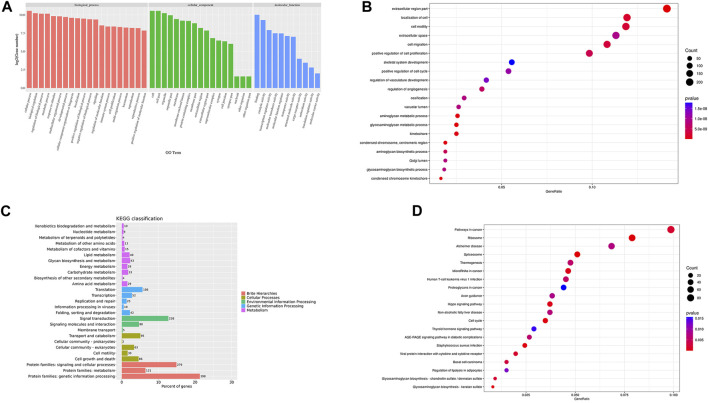
GO/KEGG cluster histogram and enrichment analysis bubble diagram Legend: **(A)**. The DEGs GO cluster histogram. **(B)**. The DEGs GO enrichment bubble diagram. **(C)**. The KEGG cluster histogram of DEGs. **(D)**. The KEGG enrichment bubble diagram of DEGs; the darker the color, the more significant the enrichment, and the larger the bubble, the larger the GeneRatio.

GO cluster analysis showed that there were 1,615 DEGs clusters related to BP, 1,584 related to CC, and 1,397 related to MF (Figure 7A). The results of GO enrichment analysis showed that in terms of BP, DEGs were enriched in the response to stimulus, the immune response, the regulation of vasculature development, cell migration, skeletal system development, the regulation of vasculature development, cell migration, and animal organ morphogenesis. In terms of CC, DEGs were enriched in the ribonucleoprotein complex, the chromosome centromeric region, condensed chromosomes, extracellular spaces, the cell surface, etc. In terms of MF, DEGs were enriched in structural constituents of the ribosome, RNA binding, Wnt protein binding, and the combination of growth factor receptors (Figure 7B).

### 3.9 The results of KEGG clustering and enrichment of DEGs

To further study the clustering and enrichment pathways of DEGs, we performed KEGG analysis. The pathways of DEGs were mainly enriched in brite hierarchies, cellular processes, environmental information processing, and genetic information processing and metabolism (Figure 7C). The DEGs were mainly enriched in pathways in cancer, the PI3K-AKT signaling pathway, the AGE-RAGE signaling pathway, *S. aureus* infection, the P53 signaling pathway,etc. (Figure 7D).

### 3.10 PPI network establishment and core module and gene identification

**FIGURE 8:**
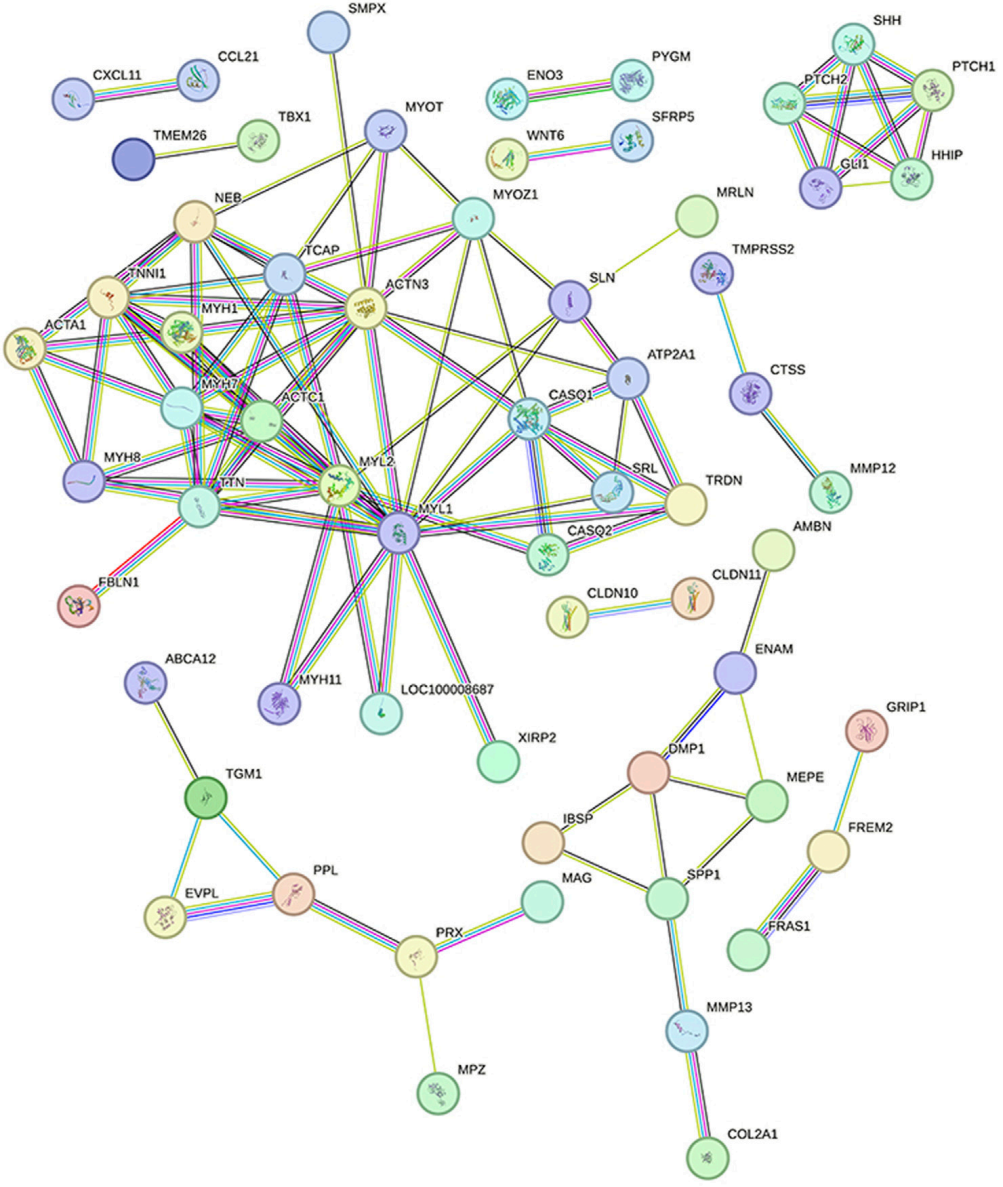
Protein interaction network diagram Legend: Each node represents the proteins produced by a single, protein-coding gene locus. The lines between nodes represent the interactions between proteins.

To determine which genes and proteins play key central roles in infection, PPI analysis of DEGs was performed using the String database. After infection, the PPI network had a total of 62 nodes and 220 functional relationships between nodes (Figure 8).

**FIGURE 9:**
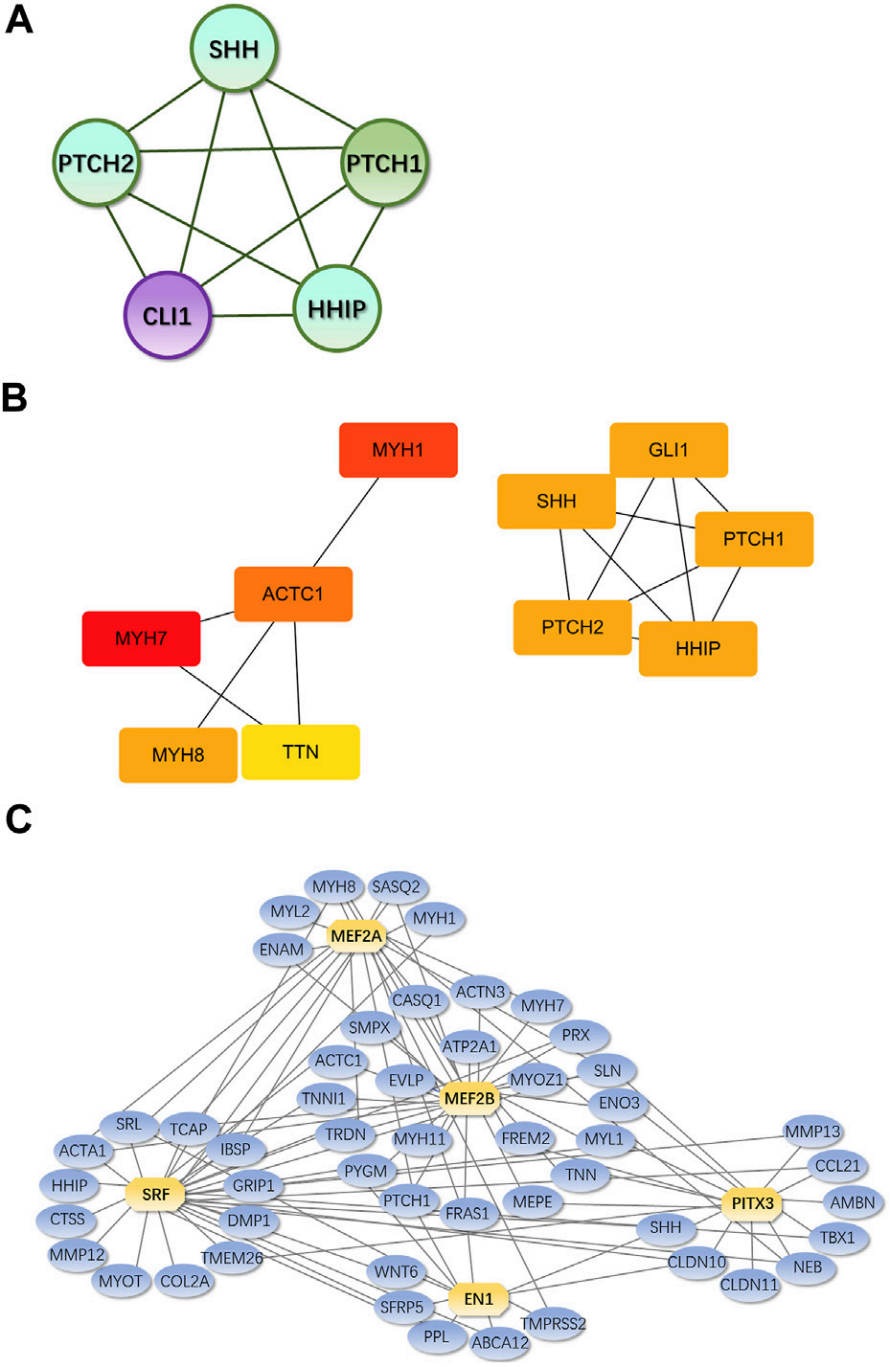
Core genes and modules and iRegulon predictions from PPI Legend: **(A)**. Core module 1. **(B)**. Core genes from cytoHubba; the darker the color, the higher the score and ranking; circles represent genes in the core module and lines represent the mutual regulation of genes. **(C)**. Transcription factors and their regulatory networks; Yellow indicates the predicted transcription factor, blue indicates DEGs, and the lines show the regulatory relationship between transcription factors and DEGs.

MCODE plug-in analysis showed that core module 1 with the highest score in the PPI network included the Hedgehog signaling pathway (Figure 9A). Further analysis by the cytoHubba plug-in showed that the core genes that met the screening criteria were patched 1, 2 (PTCH1&2), hedgehog interacting protein (HHIP), GLI1, GLI-Kruppel family member, sonic hedgehog signaling molecule (SHH), myosin heavy chain 1&7&8 (MYH1&7&8), tenascin N (TNN), and actin alpha cardiac muscle 1 (ACTC1) (Figure 9B). Among them, PTCH1&2, HHIP, GLI1 and SHH were in the first module.

### 3.11 Prediction of transcription factors and the establishment of regulatory networks

According to iRegulon predictions, transcription factors with NES>4.5 were myocyte enhancer factor 2A, 2B gene (MEF2A, MEF2B), serum response factor gene (SRF), engrailed homeobox 1 gene (EN1) and paired-like homeodomain 3 gene (PITX3), which regulated 21, 33, 31, 11, and 15 DEGs, respectively (Figure 9C).

## 4 Discussion

To explore the mechanism of mandibular infection and evaluate the effect of bone repair materials on mandibular repair, it is necessary to select a suitable animal model. Animal experiments can avoid the risks of experiments in humans; in these experiments, the conditions are controllable and reliable data can be obtained. The use of experimental animals to simulate IMD is helpful to understand the mechanism of mandibular infection and the characteristics and properties of osteogenic materials.

Beagle dogs, miniature pigs, monkeys and other large animals, whose teeth and mandible structure are similar to humans, are ideal experimental animals for modeling the dental alveolar bone and mandible (Pieri et al., 2009; Ruehe et al., 2009; Tatić et al., 2010; Hwang et al., 2019). However, large animals are more expensive, they require more specific environments, and there are few canine models, most likely due to the ethical issues associated with using animals that are typically family pets. Rabbits and mice are small and medium-sized animals that are low cost and convenient and can be used to simulate many diseases in a short period of time; thus, these are important animal models in scientific research (Freilich et al., 2009; Munhoz et al., 2011; Bayar et al., 2012; Wang et al., 2018). However, the mouse mandible is small and difficult to manipulate, and mise are often used as a model of periodontitis (de Molon et al., 2014; Liu et al., 2021). Compared with rats, rabbits have larger mandibles and thicker bone walls, which can be used to simulate IMD. X-rays showed that the mandibular ascending branch of rabbits was very thin, and the defect here could not guarantee the stability of subsequent implant materials. The root of the molar in the mandibular body is longer, which can easily damage the root when the mandibular defect is made, and the front-end edentulous jaw area will not be disturbed; thus, this model does not affect rabbit eating and is more in line with ethical guidelines. Therefore, we chose to model the edentulous jaw region of rabbits.

In war trauma, bone infection is often caused by a variety of bacteria, among which, *S. aureus* and *P. aeruginosa* have a high detection rate (Ghieh et al., 2023). Clinically, *S. aureus* infection is the most common form of bone infection, so *S. aureus* is often the first choice for the establishment of an infectious bone defect model (Lüthje et al., 2020; Watson et al., 2020; Karau et al., 2022). *P. aeruginosa* can also be found in the infections of patients with bone infection. However, most bone infection models use a single bacterial strain (Norden and Keleti, 1980), so we chose these two types of bacteria to make our model. For the amount of bacteria, domestic and foreign scholars often use 10^6^∼10^8^ CFU/mL to make the model (Wu et al., 2020; Wu et al., 2022). Due to the limitation of the scope of the defect area, we chose to inject 50 μL of a 3 × 10^8^ CFU/mL bacterial mixture. Obvious pathological changes in bone infection appeared after 14 and 28 days, and the success rate reached 100%. There was no animal death during the observation period, indicating that 3 × 10^8^ CFU/mL was the appropriate modeling concentration for the bacterial mixture. For bacterial implantation methods, most scholars utilize direct injection (Hriouech et al., 2020; Li et al., 2022; Yin et al., 2022). When the research involves implants, such as titanium implants and titanium nails, some scholars first implant bacteria on the surface of the implants and then put implants into the modeling area (Karau et al., 2022; Yang et al., 2022). Considering that the modeling simulates IMD caused by war trauma, we chose the method of directly injecting bacterial solution after the inducing mandible defects.

We used 5% sodium morrhuate as a vascular hardener, which blocks microcirculation, helps prevent systemic blood-borne infection, and simulates local blood circulation necrosis (Inzana et al., 2016), which occurs on the battlefield. Some people think that the use of vascular hardeners interferes with the establishment of infection models, but most people think that 5% sodium cod liver oleate, as an important treatment factor in the experiment, does not affect the modeling of bone infection and can prevent the failure of modeling due to macrophage phagocytosis of bacteria (Li et al., 2022; Wang et al., 2022).

There are many ways to evaluate bone infection, such as gross observation, phenotypic evaluation and histological evaluation. For this model, we observed the general condition of rabbits at 9:00 a.m. every day before and after surgery, which was consistent with what was done in previous reports. The normal body temperature of rabbits at room temperature was 38.6°C (Kasa and Thwaites, 1990). After modeling, the rabbits’ body temperature increased, food intake decreased, and local skin redness and swelling were the most intuitive signs of infection (Atkins, 1964). After modeling, the skin was red and swollen, the sinus was still present at 28 days, and pus could be seen flowing out of the sinus, proving that the infection persisted.

CBCT is one of the imaging devices that is commonly used in stomatology. Compared with X-ray, CBCT can be used to detect changes in the jaw in multiple dimensions. To dynamically observe the manifestations of bone tissue infection, we used CBCT for noninvasive detection after anesthetizing rabbits in different time. Due to the short observation time, serious symptoms such as bone malformations and pathological fractures did not appear in this model. The imaging observations were similar to the clinical findings and included osteolysis and decreased bone density (Lew and Waldvogel, 2004).

Some scholars conduct sampling and testing at 6–8 weeks (Odekerken et al., 2013; Bilgili et al., 2015), while others conduct testing at 3–4 weeks (Hwang et al., 2019; Zhang et al., 2019). The selection of model testing time depends on the type of model and the purpose of modeling. We chose 2–4 weeks because the histopathological scores at the two time points in the model have no significant difference, and the purpose of our modeling was to explore the pathogenesis of IMD to find appropriate treatment methods for early intervention. During the sampling process, pus lesions of varying degrees were found in the soft tissues around the site. After sampling, there was no periosteum covering the defect area, the bone surface was exposed, and the color was normal. The defect was filled with yellowish-white pus. This is consistent with the symptoms of animal bone limb infection models (Afzelius et al., 2022; Li et al., 2022).

Due to the influence of various factors, such as sampling site, surgical operation and bacterial virulence, there are many patients with bone infection that have negative bacterial cultures in clinical practice; thus, positive bacterial cultures are not necessary for the diagnosis of every bone infection. However, we used LB medium to culture bacteria from pus at the defect, and the results were positive. However, there was no significant change in the bacterial quantity at 28 days of infection or the ratio at 14 days of infection, indicating that the bacteria were stable in the middle and later stages, which was consistent with the findings of previous research reports (Vitko and Richardson, 2013). For the identification of bacterial species, traditional methods include observing the morphology of bacteria under an electron microscope and mass spectrometry identification. However, the observation error when using electron microscopy is large, and mass spectrometry analysis takes a long time (Alharbi et al., 2021). Existing biofluorescence imaging techniques can prevents animal sacrifice, but bacteria with fluorescent labels need to be constructed, and there is sometimes differences between the fluorescence intensity and the actual bacterial population (Kadurugamuwa et al., 2003). In recent years, PCR technology and high-throughput sequencing have emerged as strategies to detect bacteria, and these methods can accelerate the qualitative analysis of bacteria (Watts et al., 2017). We used PCR technology to compare the cultured bacteria with standard bacteria and demonstrated that the infection was caused by the two bacteria used in the modeling; furthermore, there was no interference from other bacteria during the operation and postoperative observation period.

Histopathological diagnosis is essential for bone infection, and appropriate scoring criteria are needed for complex histological changes. Smeltzer systematically proposed histopathological scoring criteria for rabbit tibial bone infection in the early stage; these criteria include aspects such as intramedullary inflammatory infiltration, intramedullary abscesses and osteonecrosis (Smeltzer et al., 1997). Currently, these aspects are used as evaluation criteria for the histopathological diagnosis of bone infection (Tiemann et al., 2014). After staining the bone tissue sections in the defect area, we used the Smeltzer score to distinguish the histopathological severity of the infection model; these results were consistent with the imaging results, and pathological changes such as inflammatory cell infiltration, bone cell disappearance, and dead bone formation occurred in the model. Goldner tricolor staining results also indicated that the number of cancellous bone trabeculae around the defect was significantly reduced, and these changes verified that this model was consistent with the clinical pathological changes seen in bone infection (Tiemann et al., 2014; Jiang et al., 2019). In our results, the pathological tissue scores between the 14-day group and the 28-day group have no significant difference, which may indicate that the infection entered a chronic stage as a result of process stabilization.

As a specific marker enzyme that stains osteoclasts, TRAP staining can be applied to observe the distribution of osteoclasts over time and determine the osteoclast count per unit area to monitor the progression of bone destruction. The results showed that osteoclasts mainly appeared and gathered around the defect, and the number of osteoclasts increased with time. Some scholars believe that bacteria can contact osteoblasts after infecting bone tissue and be internalized in cells for long-term survival, during which they cause serious damage to bone tissue (Foster et al., 2014). Although the specific mechanism of the changes in bone mass caused by complex bacteria has not been fully elucidated, the experimental results suggest that, under the condition of infection, the intramedullary cancellous bone structure may be caused by the direct action of bacteria and the induction of proinflammatory factors, which promote the function of osteoclasts and inhibit osteoblasts; this action results in the gradual absorption of cancellous bone and causes further bone loss.

Infectious bone defects have become a research hot issue in recent years, but current studies focus on the role of specific genes (Evans et al., 1998; Scianaro et al., 2014; Delsing et al., 2015; Hofmann et al., 2016b; Van Asten et al., 2017). However, the response of patients with infectious jaw defects to pathogens is systematic, and we should pay attention not only to the immune response to bacteria but also to other aspects of the response, especially bone metabolism. Many patients have a risk of bone nonunion and pathological fracture due to bone infection throughout the course of this disease. Therefore, we need to further explore the overall transcriptomic changes in bone tissue during bone infection and search for key genes to provide ideas for the early diagnosis, systematic treatment and prevention of infectious jaw defects.

Through high-throughput transcriptomic sequencing analysis, we identified 1804 DEGs when comparing the experimental group and healthy rabbits 14 days after surgery. The DEGs were enriched in BPs including immune response, the response to stimulation, the development of the bone and vascular system and the regulation of cell movement. The same trends were seen for CC and MF. According to these results, the biological function of the body after infection includes the stimulation and immune response to pathogens. Meanwhile, the expression of genes contained in angiogenesis, osteogenesis and cell migration is downregulated, and bone metabolism is inhibited.

Similar to GO analysis, KEGG pathway analysis showed that the DEGs were enriched in tumor-related pathways, the PI3k-AKT pathway, the AGE-RAGE signaling pathway, etc. Cytokines produced by chronic inflammation cause abnormal inflammatory signaling pathway activation by inducing gene mutations and altering the expression and transformation of oncogenes and tumor suppressor genes. Meanwhile, chronic inflammation promotes the establishment of an immunosuppressive tumor microenvironment by recruiting a variety of immunosuppressive cells, which promotes the occurrence and development of tumors (Guthrie et al., 2013; Schaue et al., 2015). The PI3K-AKT and AGE-RAGE signaling pathways are closely related to inflammation (Stark et al., 2015). Akt is involved in inflammation and is a key protein downstream of PI3K signaling (Testa and Tsichlis, 2005). Lipopolysaccharides (LPS) stimulate human innate immune cells, and the expression of the proinflammatory factors IL-12, TNF-α and IL-6 increases after the administration of PI3K or Akt inhibitors, while the expression of the anti-inflammatory factor IL-10 decreases (Yin et al., 2016). Studies have shown that Akt activation can inhibit LPS-induced inflammation in mice and rabbits with sepsis (Wang et al., 2013). In some patients with type II diabetes, AGEs in periodontal tissues are elevated due to increased blood glucose, and AGEs bind to RAGE on the surface of immune cells, inducing the release of inflammatory factors, and ultimately accelerating the destruction of periodontal tissues (Blasco-Baque et al., 2017; Figueredo et al., 2019). Pathway enrichment analysis showed that pathways associated with the immune system and bone destruction were activated after infection, as well as pathways associated with anti-inflammatory activity.

In the PPI network constructed based on DEGs, the modules with the top 1 scores based on the MCODE plug-in were regarded as core modules; these modules were enriched in the Hedgehog signaling pathway. It is speculated that these molecular events are related to the pathogenesis of IMD. The core genes identified by the cytoHubba plug-in were SHH GLI1, PTCH1&2, HHIP, MYH1&7&8, ACTC1 and TNN, which were all located in the center of the PPI network and were concentrated in core modules; thus, we speculate that these genes may be intervention targets for repairing IMD.

Among the identified core genes, SHH, PTCH1&2, HHIP, and GLI1 are components of the Hedgehog signaling pathway, which is closely related to inflammation and tissue repair. Lowrey et al. (Lowrey et al., 2002) showed that when exogenous SHH peptide is added to the Hedgehog signaling pathway, and the proliferation of anti-CD3/CD28-activated peripheral blood CD4^+^ T cells significantly increased. Kim JH et al. (Kim et al., 2010) showed that *Helicobacter pylori* can activate NF-kB and induce SHH expression in a CAGa-dependent manner, and SHH can be used as a chemical inducer of macrophages to recruit macrophages to the inflammatory region (Schumacher et al., 2012). In addition, SHH is also a chemical inducer of BMSCS in chronic inflammation (Warzecha et al., 2006). Circulatory signals (such as TGF-β) released during H. pylori-mediated gastritis can induce HH/GLI signaling in bone marrow-derived stromal cells and enable BMSCS recruitment to the inflammatory region. Cai et al. (Cai et al., 2012) interfered with rat BMSCs using rShh-N and found that rShh-N increased the proportion of cells in the S phase and the G2/M phase, enhanced ALP activity and matrix mineralization ability, and increased the expression of osteogenic genes, indicating that SHH promotes the proliferation and osteogenic differentiation of BMSCs. Baht et al. (Baht et al., 2014) used the mouse tibial fracture model and found that the expression of the Hedgehog target genes (GLI1 and PTC1) gradually increased 7, 14, and 21 days after fracture repair in wild-type mice, indicating that the activation of Hedgehog signaling plays a role in regulating fracture healing. In conclusion, the Hedgehog signaling pathway is involved in regulating the immune system during infection and can recruit BMSCS cells to accelerate repair in the body. Therefore, the intervention of this pathway is expected to control infection and promote the repair of mandibular defects.

In this study, the MEF2 family was the transcription factor family whose prediction results were of the highest confidence in the IRegulon, which is consistent with the results of Zhang RK (Zhang et al., 2018). MEF2A is a component of the MEF2 transcription factor family (Pon and Marra, 2016). Chen C’s (Chen et al., 2023) study indicated that Mef2A may upregulate the expression of the collagen X gene (Col10a1) by interacting with its cis-enhancer, and changes in MEF2A levels affect the chondrogenic marker gene’s expression, such as Runx2 and Sox9. Blixt N (Blixt et al., 2020) showed that the loss of Mef2A inhibited the differentiation and activity of osteoclasts by altering the expression of key proteins that regulate the expression of osteoclast genes, and the ability of osteoclasts to differentiate was almost completely inhibited. The regulation of this transcription factor is expected to inhibit the activity of osteoclasts and prevent further expansion of bone defects.

In summary, we successfully established an infection defect animal model of the mandibular, which was formed by directly injecting bacterial solution into the defect area. The establishment process for this model was relatively simple, the production cost was low, the method has strong repeatability, and this model can simulate the histopathological changes seen in clinical bone infections. This model is a basic tool for the study of mandibular defects infected with complex bacteria. Subsequently, we screened and identified susceptibility genes, transcription factors and related signaling pathways associated with IMD by transcriptome sequencing and bioinformatics analysis. A total of 1804 DEGs related to IMD were found, including 11 core genes and 1 key transcription factor family. The above signaling pathways involved in the occurrence of IMD include immune regulation, promoting osteogenesis and regulating osteoclast differentiation. The treatment of infectious mandibular defects requires not only infection control, but also restoration of mandibular defects caused by infection. Therefore, further study of these molecules and their pathways will help to elucidate the specific mechanism of IMD and discover the target of repair IMD related to the regulation of immunity, the promotion of osteogenesis and the inhibition of osteoclast.

## Data Availability

The datasets presented in this study can be found in online repositories. The names of the repository/repositories and accession number(s) can be found below: https://www.ncbi.nlm.nih.gov/, GSE248915.
